# Real-world safety profile of live attenuated Japanese encephalitis vaccine before and after the vaccine administration law

**DOI:** 10.1371/journal.pone.0326257

**Published:** 2025-06-16

**Authors:** Chao Zhang, Yan Liu, Xiaofu Luo, Liping Han, Jianyong Shen

**Affiliations:** Huzhou Center for Disease Control and Prevention, Huzhou, China; Mahidol Oxford Clinical Research Unitl (MORU), THAILAND

## Abstract

**Purpose:**

Live attenuated Japanese encephalitis vaccine (JEV-L) was included in the Expanded program on immunization on Immunization (EPI) in 2008 and has had a satisfactory effectiveness and safety profile in protecting children from JE virus infection. This study was designed to evaluate the surveillance sensitivity and changes over time of adverse event following immunization (AEFI) reports related to JEV-L based on the national AEFI surveillance system(NAEFISS) data before and after the introduction of vaccine administration law (LAW) from 2014 to2023 in Huzhou City.

**Methods:**

AEFI data were collected from NAEFISS from 2014 to 2023 and included age, AEFI and diagnostic categories. AEFI incidence rates are calculated/10,000 vaccine doses and a reporting odds ratio −1.96 standard error (ROR-1.96SE) >1 defined a positive signal between AEFI and vaccine. We categorized the time interval into the pre-LAW period (2014–2019) and post-LAW period (2020–2023) to demonstrate the impact of LAW on the surveillance of AEFI.

**Results:**

The NAEFISS collected 225 AEFI reports after administering 599,223 doses of JEV-L, with a AEFI reported rate of 3.75/10,000 doses. Overall AEFI reported rates increased over time with a drop in 2020 (P < 0.001, χ2 for trend). The reported rate of AEFI in post-LAW period was higher than that in the pre-LAW period (P < 0.001), accounted for increases in fever, local redness and local induration. However, the reported rate of urticaria decreased significantly in post-LAW period(P = 0.043). This study found a positive signal association between JEV-L immunization and fever in the pre- (1.394) and post-LAW period(2.833) and a signal for urticaria in the pre-LAW period (2.098). There were only 2 severe reactions reported: thrombocytopenia and epilepsy.

**Conclusions:**

The implementation of the LAW significantly enhanced the surveillance capacity and sensitivity for AEFIs associated with JEV-L. This study did not find any new/unexpected safety concern in the post-LAW period. Severe reactions remain rare despite the greatly improved sensitivity of surveillance, indicating that the vaccine was comparatively safe. However, continuous epidemiological investigation are needed to systematically assessment the data provided by NAEFISS.

## Introduction

Japanese encephalitis (JE), caused by the mosquito-borne flavivirus JEV, represents a significant public health threat in Asia, with approximately 3.4 billion people at risk, including 700 million children [1–3]. JEV represents the predominant cause of pediatric viral encephalitis in Asia, particularly affecting children under 15 years [4], with rising incidence observed among adults in recent years in China [5]. JE is one of the major public health problems threatening population health in Asia and the Western Pacific [6].

While numerous viruses (≥100 species) can cause encephalitis, regional surveillance reveals distinct etiological patterns. In Zhejiang Province, enteroviruses (32.16%) predominate in pediatric viral encephalitis cases, followed by Epstein–Barr virus(EBV) (3.96%) and human herpesvirus 6(HHV-6) (2.64%) [7]. It is worth noting that although JEV is not the main pathogen of viral encephalitis in Zhejiang Province, JE has become an important public health problem in the world, especially in Southeast Asia and the Western Pacific region because of its high mortality and high disability rate.

China currently employs two Japanese encephalitis vaccines: the live-attenuated vaccine (JE-L), introduced in 1989 and included into Expanded Program on Immunization (EPI) in 2008 [8], and the inactivated JE vaccine (JE-I) was developed in China and has been used since the 1970s as a non-EPI vaccine option [9]. The incidence of JE has remained within 0.3/100,000 in China since the widespread implementation of JEV-L vaccination [10,11]. JE in China occurs mainly in rural areas. Studies showed that The Yangtze River Plain is typical of large-scale farmland irrigation, which also facilitates the natural circulation of JE, making this region one of the regions with the largest proportion of JE cases in China [12]. According to the JE surveillance data, the average annual incidence was 0.10/100,000 in Huzhou from 2007 to 2023, and no JE case was reported after 2019 [13]. While vaccine-preventable diseases have been effectively controlled in China, heightened surveillance has revealed increasing reports of adverse events following immunization (AEFI) [14]. This trend primarily reflects the AEFI surveillance has been greatly improved after monitoring systems established in 2005 [15], rather than actual increases in vaccine risks. Such enhanced detection, while crucial for vaccine safety monitoring and public confidence [16,17], has paradoxically amplified safety concerns among vaccine recipients despite high JEV-L coverage rates. Systematic evaluation of JEV-L-associated AEFI is therefore critical for maintaining public trust and guiding national immunization policy.

Recent vaccine safety incidents, notably the 2018 Changchun Changsheng event, have markedly eroded public trust in immunization programs [18,19]. Chinese vaccine administration law(LAW) is a law specifically aimed at vaccine management, and its promulgation has a direct relationship with vaccine-related events. The implementation of the Law has standardized vaccination evaluation and management systems [20]. However, limited data exist regarding its impact on AEFI surveillance, particularly for the widely-used JEV-L In this study, we assess the reporting rate of AEFI related to JEV-L from 2014 to 2023 in Huzhou city based on real- world data derived from AEFI surveillance system before and after the LAW. This study aimed to provide evidence to support the public perception of the safety of this vaccine, offer sensitivity analysis of the passive surveillance of AEFIs before and after the LAW, and hence increase public confidence in JEV-L vaccination.

## Materials and methods

### Sources of AEFI data

The AEFI reports following JEV-L from 2014 to 2023 were extracted from the national adverse event following immunization surveillance system(NAEFISS) [21]. NAEFISS is a national passive surveillance system to detect common and rare AEFIs by collecting AEFI reports nationwide The national surveillance system included variables of relevant attributes such as age, date of vaccination, outcome, and so on. The number of JEV-L doses during the same period was obtained from the national immunization program vaccine use report. The live attenuated JE vaccine (SA 14-14-2 strain, Chengdu Institute of Biological Products and Wuhan Institute of Biological Products) was exclusively used during surveillance.

### Study design

The descriptive analyses of NAEFISS data were conducted to calculate the JEV-L reporting. We categorized the time interval into the pre-LAW period (2014–2019) and the post-LAW period (2020–2023) to demonstrate the impact of LAW on the surveillance of AEFI following vaccination with JEV-L.

### Category of AEFI

All AEFI reports are divided into vaccine product-related reaction(common and rare vaccine reaction), coincidental event, psychogenic reaction, vaccine quality event and program error according to the National Monitoring Program for Suspected Abnormal Reaction of Vaccination [22]. AEFI to be reported after vaccination include, but are not limited to, fever(≥38. 6°C), local redness(>2.5 cm), induration(>2.5 cm), allergic rash (including urticaria, maculopapules, and measles scarlet fever-like rash), allergic shock, allergic laryngeal edema, angioedema, allergic purpura, thrombocytopenic purpura, localized allergic necrotic reaction (Arthus reaction), febrile convulsion, encephalitis and meningitis, epilepsy, brachial neuritis, Guillain-Barré syndrome and so on. Common vaccine reaction: the reaction that occurs after vaccination and is caused by the inherent characteristics of the vaccine itself, which will only cause transient physiological dysfunction to the body, mainly including fever, local redness and local induration in this study. Rare vaccine reaction refers to the qualified vaccine causes damage to the tissues, organs, and functions of the recipient during or after the implementation of the standard vaccination, and the relevant parties are not at fault, mainly including allergic reaction, nervous system reaction, and other reactions in this study. AEFI severities were assessed as two types, serious and non-serious: (1) non-serious, with no additional intervention or with hospital visit or event interfering with daily activities or loss of working hours. (2) serious, serious, with any untoward medical occurrence that results in death, hospitalization, prolongation of hospitalization, persistent or significant disability/incapacity, life threatening or birth defect.

### Data analysis

The AEFI datas of JEV-L were collected using Microsoft Office Excel 2020, and descriptive analysis was conducted using R software 4.4.2. The characteristics of reports were summarized by category, severity, patient age, and interval of AEFI onset, clinical diagnosis. The AEFI rate was calculated per 10,000 doses of JEV-L administered. The average incidence rate of AEFI following JEV-L vaccination in the pre-LAW period (2014–2019) and post-LAW period (2020−2023) were calculated. Inter-group rate comparisons were conducted using the chi-square test, and the Chi-squared trend test was used to analyse the trend of report rates. Reporting odds ratio(ROR) was used to detect the abnormal response signals in the clinical diagnosis of JEV-L with a total of three or more cases of abnormal reaction from 2014 to 2023 in this study. A value of ROR-1.96SE > 1 (standard error [SE]) is considered a positive signal [23].

## Results

### Basic characteristic of AEFI reports

**Fig 1:**
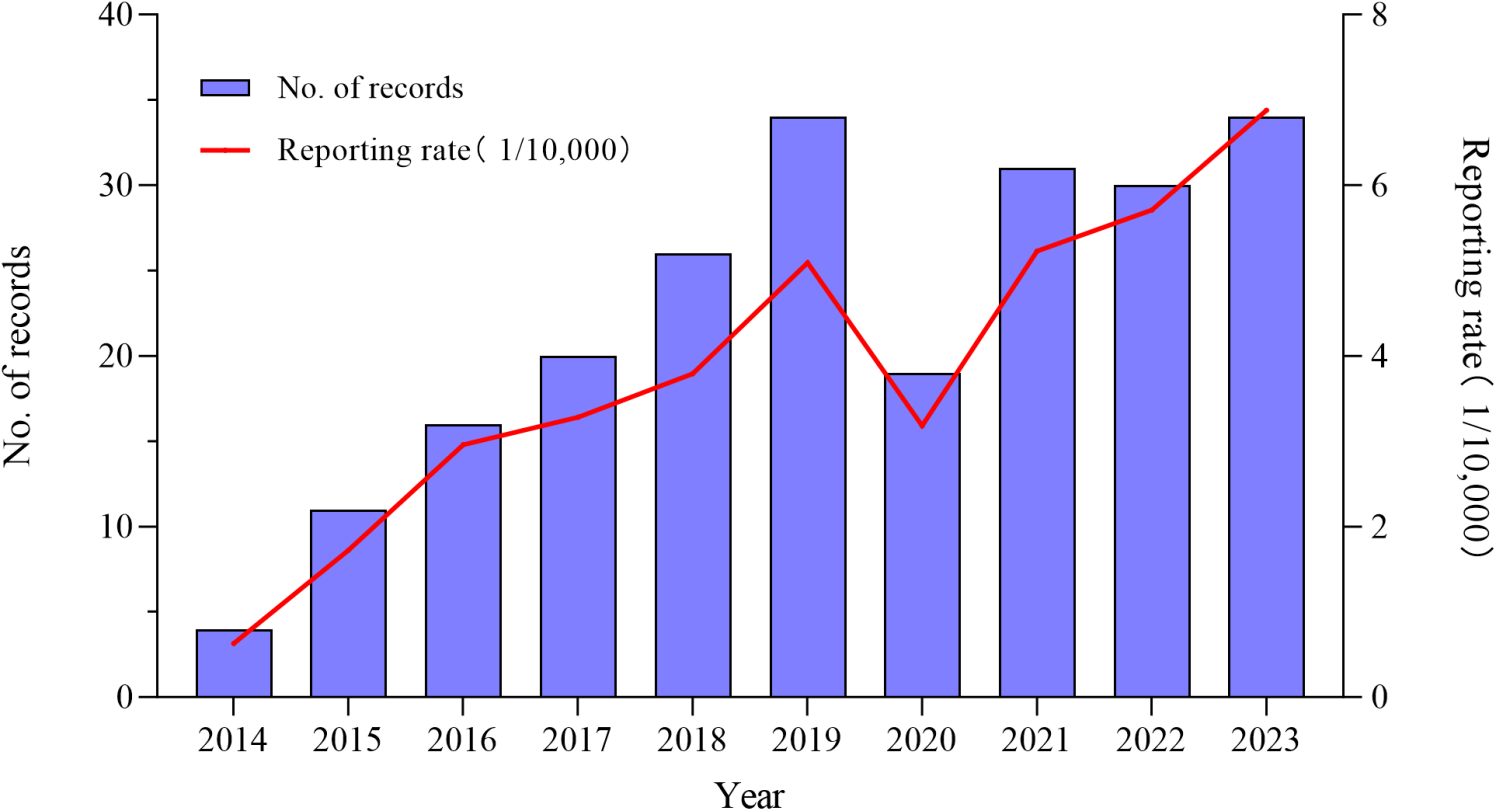
Reporting rate of AEFI of JEV-L in Huzhou, Zhejiang province, 2014–2023 (/10,000).

From 2014 to 2023, The NAEFISS collected 225 AEFI reports after administering 599,223 doses of JEV-L, with a AEFI reported rate of 3.75/10,000 doses. The annual reported rate of AEFI increased from 0.63/10000 to 6.88/10,000 over this period in total. The overall AEFI reported rate was on the rise(χ^2^ = 43.401, P < 0.001), but with a drop in 2020 (3.18/10,000) (Fig 1). Of which, 223 cases (99.11%, 3.72/10,000 doses) were classified as non-serious, while 2 (0.89%, 0.03/10,000 doses) were classified as serious(thrombocytopenic purpura and epilepsy).

In this study, 223 cases of vaccine product-related reactions and 2 cases of coincidental events were reported, and the reported rate was 3.72/10,000(223/599,223) and 0.03/10,000(2/599,223), respectively. There were 109 and 114 cases of vaccine product-related reactions reported in the pre-LAW and post-LAW period, respectively. 109 cases(2.88/10,000) of vaccine product-related reactions were reported in the pre-LAW period and 114 cases(5.16/10,000) were reported in the post-LAW period. 2 cases of coincidental events(0.05/10,000) were reported in the pre-LAW period, and no coincidental event were reported in the post-LAW period.

The highest reported rate was fever(2.77/10,000), which was 2.88/10000 in the pre-LAW period and 5.16/10000 in the post-LAW period. The reported rate of local redness and local induration were 0.85/10000(0.56/10,000 in the pre-LAW period and 1.36/10,000 in the post-LAW period) and 0.25/10000(0.05/10,000 in the pre-LAW period and 0.59/10,000 in the post-LAW period), respectively. In the pre-LAW period, the reported rate of anaphylaxis, rash, allergic rash, urticaria, maculopapular rash, thrombocytopenia purpura and epilepsy was 0.71/10,000, 0.71/10,000, 0.34/10,000, 0.34/10,000, 0.03/10,000, 0.03/10,000 and 0.03/10,000, respectively. In the post-LAW period, the reported rate of anaphylaxis, rash, allergic rash and urticaria was 0.36/10,000, 0.36/10,000, 0.32/10,000 and 0.05/10,000, respectively. No cases of maculopapular rash, thrombocytopenia purpura and epilepsy were reported in the post-LAW period.

The majority of AEFI reports were observed aged 1–4years (52.25% in the pre-LAW period and 51.75% in the post LAW period) and <1 year (46.85% in the pre-LAW period and 47.37% in the post LAW period). 5–9 years accounted for 0.90% in the pre-LAW period and 0.88% in the post-LAW period. The time interval of most symptoms onset from vaccination was within 24 hours (93.69% in the pre-LAW period and 91.23% in the post LAW period) (Table 1).

**Table 1:** Characteristics of AEFIs with JEV-L vaccine in the pre-LAW and post-LAW period.

Parameter	Pre LAW			Post LAW		
	No.	Denominator	Rate	No.	Denominator	Rate
*Program classification of events(1/10,000)* ^a^						
Vaccine Product-related Reaction	109	378,358	2.88	114	220,865	5.16
Vaccine Quality Event	0	378,358	0.00	0	220,865	0.00
Program Error	0	378,358	0.00	0	220,865	0.00
Coincidental Event	2	378,358	0.05	0	220,865	0.00
Psychogenic Reaction	0	378,358	0.00	0	220,865	0.00
*Individual adverse events(1/10,000)* ^a^						
Fever	77	378,358	2.04	89	220,865	4.03
Anaphylaxis	27	378,358	0.71	8	220,865	0.36
Urticaria	13	378,358	0.34	1	220,865	0.05
maculopapular rash	1	378,375	0.03	0	220865	0.00
allergic rash	13	378,375	0.34	7	220865	0.32
Rash^*^	27	378,358	0.71	8	220,865	0.36
local redness	21	378,358	0.56	30	220,865	1.36
local induration	2	378,358	0.05	13	220,865	0.59
Thrombocytopenia purpura	1	378,358	0.03	0	220,865	0.00
Epilepsy	1	378,358	0.03	0	220,865	0.00
*Age groups in years(%)* ^b^						
<1	52	111	46.85	54	114	47.37
1–4	58	111	52.25	59	114	51.75
5–9	1	111	0.90	1	114	0.88
*Timing of AE in days(%)* ^b^						
≤ 1	104	111	93.69	104	114	91.23
2–3	6	111	5.41	7	114	6.14
4–7	1	111	0.90	2	114	1.75
>7	0	111	0.00	1	114	0.88

* Include allergic rash (20 cases), urticaria (14 cases) and maculopapular rash (1 case).

^a^Parameters were expressed as reported incidence rate.

^b^Parameters were expressed as constituent ratio.

### Changes in the reported rates of AEFIs between pre-LAW period and LAW period

The average reported rate of total AEFIs of the LAW period was 5.16/10,000, which was dramatically higher than pre-LAW period (2.93/100000), and the difference was statistically significant (χ^2^ = 17.851, P = 0.000). Rates of vaccine product-related reactions in the post-LAW period(5.16/10,000) was higher than that in the pre-LAW (2.88/10,000), and the difference was statistically significant (χ^2^ = 18.891, P < 0.001) (Table 2).

**Table 2:** Changes in reported rate of AEFIs in the pre-LAW and post-LAW period(/10000).

Time interval	JEV-L doses administered	Total AEFIs		Vaccine product-related reaction		Coincidental Event	
		No.	Rate	No.	Rate	No.	rate
Pre-LAW	378358	111	2.93	109	2.88	2	0.05
Post-LAW	220865	114	5.16	114	5.16	0	0.00
χ^2^			17.851		18.891		0.121
P			<0.001		<0.001		0.728

### Clinical diagnosis and abnormal signals

After reviewing the clinical diagnosis and classifications of the 225 AEFI records. The reported rates of fever, local redness, and local induration in the post-LAW period were higher than those in the pre-LAW period(P < 0.05). However, the reported rate of urticaria in the post-LAW period was lower than that in the pre-LAW period(P = 0.043). The abnormal signals with according to ROR-1.96SE were detected for fever(1.394), urticaria(2.098) and rash(1.056) in the pre-LAW period, and positive signals were obtained for fever(2.833) in the post-LAW period (Table 3).

**Table 3:** Clinical diagnosis and abnormal signals of AEFIs(/10,000).

Clinical diagnosis	Pre-LAW period			Post-LAW period			*χ* ^*2*^	*P*
	No.	Rate	ROR-1.96SE	No.	Rate	ROR-1.96SE		
Fever	77	2.04	1.394	89	4.03	2.833	19.318	<0.001
Local redness	21	0.56	0.207	30	1.36	0.304	9.650	0.002
local induration	2	0.05	0.019	13	0.59	0.239	13.921	<0.001
Anaphylaxis	27	0.71	0.988	8	0.36	0.338	2.377	0.123
Urticaria	13	0.34	2.098	1	0.05	0.108	4.112	0.043
Allergic rash	13	0.34	0.716	7	0.32	0.403	0.000	1.000
Rash	27	0.71	1.056	8	0.36	0.368	2.377	0.123

## Discussion

Since the inclusion of the JEV-L in China’s Expanded Program on Immunization, Huzhou city has consistently maintained high vaccination coverage. However, as JE incidence declines and JEV-L administration increases, growing attention has been paid to vaccine safety.

This review focuses on the safety of the most commonly used JEV-L in Huzhou city from 2014 to 2023. The actual number of doses of JEV-L vaccine administered per year in Huzhou city from 2014 to 2023 was used to calculate the reporting rate of AEFI. Monitoring data in this study showed that the total reported rate of JEV-L AEFI in Huzhou during 2014−2023 was 3.75/10,000 doses, which was higher than that of the whole China(estimated reported rate 96.55 per million doses) during 2009–2012 [24], but comparable to the rate of 37.14 per 100,000 doses observed in Zhejiang Province in 2019 [25]. However, the reported incidence is higher than that of live-attenuated recombinant Japanese encephalitis vaccine reported in Taiwan [26]. An increasing trend was observed in the reporting rate of AEFI for JEV-L, which is related to the generally increased sensitivity of the AEFI surveillance [27]. However, there was a decrease in the reported incidence of AEFI in 2020, which may be related to the impact of COVID-19 in 2020, such as parents avoiding medical visits for fear of mild reactions to infection, thus reducing the reporting of AEFI.

We found that the overall incidence of AEFI reports in the post-LAW period was significantly higher than that in the pre-LAW period, and the incidence of vaccine product-related reaction reports increased significantly in the post-LAW period. This significant increase was not due to the decrease in vaccine safety, but to the significantly improved sensitivity of the surveillance system. They are generally a political reflection of the vaccine incidents that occurred during this period, and also a result of the increasing public concern for vaccine safety. For example, the Vaccine Administration Act regulates the reporting process of AEFI, thereby reducing the occurrence of underreporting. where implementation of the FDA Amendments Act of 2007 was followed by a sustained increase in adverse event reporting rates for immunizations in subsequent years [28,29]. Fever, local redness and local induration were the main AEFIs, which are considered to be part of the immune response [30]. Surveillance data demonstrated a significant increase in the reporting rates of these minor adverse events in the post-LAW period. indicating substantially improved detection sensitivity for minor adverse events following legislative implementation. Some allergic reactions have been reported by surveillance systems, but the reported rate was less than 1/10,000. Most of allergic reactions were allergic rash, followed by urticaria and maculopapular rash. The results were consistent with the safety studies of JEV-L in Guangdong, China and Cambodia [31,32]. In our study, the reported rates of these allergic reactions decreased and no maculopapular rash was reported in the post-LAW period. These data further validate the safety of the JEV-L vaccine. The causes of allergic rash are complex. Different vaccine components, including various antibiotics, preservatives, stabilizers, adjuvants and other additives, as well as latex contamination, may be allergen-induced rash. IgE-mediated immediate allergic reaction is the main mechanism of allergic rash, and the corresponding symptoms usually appear 1–6 hours after exposure to the allergen [33]. The proportion of AEFI within 1 day after vaccination was more than 90% in the pre-LAW period and in the post-LAW period. It is suggested that the JEV-L vaccine recipients or their guardians should be informed about the basic knowledge of JEV-L vaccine, and close attention should be paid to the situation of the vaccine recipients within 1 day after vaccination. They should seek medical attention as soon as possible if suspicious conditions are found. In the two period, the proportion of AEFI in children younger than 1 year old and 1–4 years old was much higher than that in children aged 5–9 years. The age distribution of AEFI was consistent with the JEV-L immunization schedule in China for EPI vaccines(JEV-L vaccine is typically administered at 8 months and 2 years of age). It may also be related to parents’ more attention to infant AEFI and higher reporting initiative.

Two serious AEFI included in the NAEFISS(thrombocytopenic purpura and epilepsy) at a rate of 0.03 per 10,000 doses distributed, which only accounted for 0.89% of all reported AEFIs. The rate of serious AEFIs was similar to that of live-attenuated recombinant Japanese encephalitis vaccine in Taiwan(0.37 per 100,000 doses) [26]. This finding corroborates the safety profile of the JEV-L vaccine, demonstrating an exceptionally low risk of serious AEFIs. Moreover, abnormal signals analysis suggested that this study did not identify any new emerging safety concerns in the post-LAW period. The safety of live attenuated JE vaccine has been reconfirmed in a situation where the law has improved the sensitivity of surveillance. Nonetheless, while strengthening the sensitivity of surveillance, further studies are needed to confirm the causal relationship between live attenuated JE vaccine and these diseases, so as to provide higher confidence in these safety conclusions.

A few limitations should be acknowledged in this study, such as the data of this study came from a passive surveillance system of AEFI that is susceptible to underreporting, reporting biases, and data completeness, which may affect the accuracy of AEFI reported incidence estimates. For example, the AEFI monitoring system mandated the reporting of fever, local redness, and local induration as compulsory parameters. While additional symptoms such as fatigue, drowsiness, inappetence and so on could be documented voluntarily, these symptoms are not mandated for recording by the surveillance system. This inherent design characteristic introduced certain limitations, potentially resulting in incomplete symptom documentation. Furthermore, some cases may have presented with multiple concurrent symptoms which were simply recorded as “fever/redness/induration” in the system, and no further distinction was made between the severity of fever and pain. Underreporting of severity grading underscores the need for standardized training of AEFI reporters. However, passive surveillance is still the best and most feasible method for AEFI monitoring despite its limitations. The positive signals inability to assess causality between receipt of a vaccine and an AEFI. Continuous epidemiological investigation are needed to systematically assessment the relationships. However, there are also some strengths to this study; all AEFI cases were reported in a standardized manner in accordance with national monitoring program. Moreover, we did not find a significant increase in the rates of rare reaction during LAW period, which indicate that the rare response after JEV-L remains stable and rare, and the results are benefit to strengthen the public confidence in JEV-L vaccination.

## Conclusion

The implementation of the LAW significantly enhanced the surveillance capacity and sensitivity for AEFIs associated with JEV-L. The present study did not find any new/unexpected safety concern in the post-LAW period. Severe reactions remain rare despite the greatly improved sensitivity of surveillance, and no cases of anaphylactic shock, anaphylactic laryngeal edema, or encephalitis have been reported, indicating that the vaccine was comparatively safe. However, continuous epidemiological investigation are needed to systematically assessment the data provided by NAEFISS.

## Supporting information

S1 File Data. (XLS)

## References

[1] Ngwe Tun MM, Kyaw AK, Nwe KM, Inoue S, Thant KZ, Morita K. Effectiveness of the SA 14-14-2 live-attenuated Japanese encephalitis vaccine in Myanmar. Vaccines (Basel). 2021;9(6):568. doi: 10.3390/vaccines9060568 34072933 PMC8227667

[2] Nealon J, Taurel A-F, Yoksan S, Moureau A, Bonaparte M, Quang LC, et al. Serological evidence of Japanese encephalitis virus circulation in Asian children from dengue-endemic countries. J Infect Dis. 2019;219(3):375–81. doi: 10.1093/infdis/jiy513 30165664 PMC6325342

[3] WHO. Japanese encephalitis vaccines: WHO position paper, February 2015--recommendations. Vaccine. 2016;34(3):302–3. doi: 10.1016/j.vaccine.2015.07.057 26232543

[4] Preethi L, Alina MS, Chandran L, Asvin S, Jagadeesan M, Vijayakumar TM, et al. Duration of seroprotection of the live attenuated SA-14-14-2 Japanese encephalitis vaccine in children in India. J Travel Med. 2023;30(2):taac147. doi: 10.1093/jtm/taac147 36495206

[5] Wang L-P, Yuan Y, Liu Y-L, Lu Q-B, Shi L-S, Ren X, et al. Etiological and epidemiological features of acute meningitis or encephalitis in China: a nationwide active surveillance study. Lancet Reg Health West Pac. 2022;20:100361. doi: 10.1016/j.lanwpc.2021.100361 35036977 PMC8743210

[6] Heffelfinger JD, Li X, Batmunkh N, Grabovac V, Diorditsa S, Liyanage JB, et al. Japanese Encephalitis Surveillance and Immunization - Asia and Western Pacific Regions, 2016. MMWR Morb Mortal Wkly Rep. 2017;66(22):579–83. doi: 10.15585/mmwr.mm6622a3 28594790 PMC5720240

[7] Liu J-J, Teng L-P, Hua C-Z, Xie Y-P, Pan Y-X, Hu B-F, et al. Etiological analysis of viral encephalitis in children in Zhejiang province from 2018 to 2019. Diagnostics (Basel). 2022;12(8):1964. doi: 10.3390/diagnostics12081964 36010314 PMC9407060

[8] Deng X, Yan J-Y, He H-Q, Yan R, Sun Y, Tang X-W, et al. Serological and molecular epidemiology of Japanese Encephalitis in Zhejiang, China, 2015-2018. PLoS Negl Trop Dis. 2020;14(8):e0008574. doi: 10.1371/journal.pntd.0008574 32853274 PMC7491720

[9] Wu W, Liu D, Li K, Nuorti JP, Nohynek HM, Xu D, et al. Post-marketing safety surveillance for inactivated and live-attenuated Japanese encephalitis vaccines in China, 2008-2013. Vaccine. 2017;35(29):3666–71. doi: 10.1016/j.vaccine.2017.05.021 28552510

[10] Chen XJ, Wang HY, Li XL, Gao XY, Li MH, Fu SH, et al. Japanese Encephalitis in China in the period of 1950-2018: from discovery to control. Biomed Environ Sci. 2021;34(3):175–83. doi: 10.3967/bes2021.024 33766213

[11] Zhang H, Wang Y, Li K, Mehmood K, Gui R, Li J. Epidemiology of Japanese Encephalitis in China (2004-2015). Travel Med Infect Dis. 2019;28:109–10. doi: 10.1016/j.tmaid.2018.09.011 30267769

[12] Ren X, Fu S, Dai P, Wang H, Li Y, Li X, et al. Pigsties near dwellings as a potential risk factor for the prevalence of Japanese encephalitis virus in adult in Shanxi, China. Infect Dis Poverty. 2017;6(1):100. doi: 10.1186/s40249-017-0312-4 28592296 PMC5463306

[13] Zhang C, Shen J, Luo X, Liu Y, Han L, et al. Epidemiological characteristics of epidemic encephalitis B in Huzhou City from 2007 to 2023. China Prev Med J. 2025;37(4):386–9.

[14] Suragh TA, Lamprianou S, MacDonald NE, Loharikar AR, Balakrishnan MR, Benes O, et al. Cluster anxiety-related adverse events following immunization (AEFI): An assessment of reports detected in social media and those identified using an online search engine. Vaccine. 2018;36(40):5949–54. doi: 10.1016/j.vaccine.2018.08.064 30172632 PMC6534132

[15] Hu Y, Pan X, Chen F, Wang Y, Liang H, Shen L, et al. Surveillance of adverse events following immunization of 13-valent pneumococcal conjugate vaccine among infants, in Zhejiang province, China. Hum Vaccin Immunother. 2022;18(1):2035141. doi: 10.1080/21645515.2022.2035141 35240930 PMC9009923

[16] Chen F, Pan X, Liang H, Shen L, Wang Y, Chen Y, et al. Real-world safety profile of the 9-valent human papillomavirus vaccine: A study in Zhejiang, China from 2019 to 2021. Hum Vaccin Immunother. 2022;18(7):2152256. doi: 10.1080/21645515.2022.2152256 36484114 PMC9762803

[17] Wang C, Huang N, Lu Q-B, Black S, Liang X, Cui F. Change in adverse event reporting following immunization of hepatitis B vaccine among infants between 2013 to 2020 before and after the vaccine administration law in China. Front Immunol. 2022;13:956473. doi: 10.3389/fimmu.2022.956473 36248783 PMC9561938

[18] Hou Z, Lai X, Liu Y, Jit M, Larson HJ, Fang H. Assessing the impact of the 2018 Changchun Changsheng vaccine incident on childhood vaccination in China. Commun Med (Lond). 2023;3(1):114. doi: 10.1038/s43856-023-00339-0 37608146 PMC10444794

[19] Yu W, Cao L, Liu Y, Li K, Rodewald L, Zhang G, et al. Two media-reported vaccine events in China from 2013 to 2016: Impact on confidence and vaccine utilization. Vaccine. 2020;38(34):5541–7. doi: 10.1016/j.vaccine.2020.05.014 32620373

[20] Liu X, Yu W, Yin Z, Rodewald L, Song Y, Zhang Z, et al. Vaccine events raising public concern and associated immunization program policy and practice changes, China, 2005-2021. Vaccine. 2022;40(18):2561–7. doi: 10.1016/j.vaccine.2022.03.035 35339307

[21] Liu D, Wu W, Li K, Xu D, Ye J, Li L, et al. Surveillance of adverse events following immunization in China: Past, present, and future. Vaccine. 2015;33(32):4041–6. doi: 10.1016/j.vaccine.2015.04.060 25936727

[22] Ren M, Li K, Li Y, Fan C, Xu Y, Zhang L, et al. Post-marketing surveillance of adverse events following immunization with Haemophilus influenzae type B conjugate vaccine - China, 2010-2021. China CDC Wkly. 2024;6(33):834–40. doi: 10.46234/ccdcw2024.180 39211439 PMC11350236

[23] Hu Y, Pan X, Shen L, Chen F, Wang Y, Liang H, et al. Post-licensure safety monitoring of quadrivalent human papillomavirus vaccine using the national adverse event following immunization surveillance system from Zhejiang province, 2018-2020. Hum Vaccin Immunother. 2021;17(12):5447–53. doi: 10.1080/21645515.2021.1978793 34613883 PMC8903994

[24] Wang Y, Dong D, Cheng G, Zuo S, Liu D, Du X. Post-marketing surveillance of live-attenuated Japanese encephalitis vaccine safety in China. Vaccine. 2014;32(44):5875–9. doi: 10.1016/j.vaccine.2014.08.001 25173477

[25] Pan X, Lv H, Chen F, Wang Y, Liang H, Shen L, et al. Analysis of adverse events following immunization in Zhejiang, China, 2019: a retrospective cross-sectional study based on the passive surveillance system. Hum Vaccin Immunother. 2021;17(10):3823–30. doi: 10.1080/21645515.2021.1939621 34170800 PMC8437471

[26] Ma H-Y, Lai C-C, Chiu N-C, Lee P-I. Adverse events following immunization with the live-attenuated recombinant Japanese encephalitis vaccine (IMOJEV®) in Taiwan, 2017-18. Vaccine. 2020;38(33):5219–22. doi: 10.1016/j.vaccine.2020.06.008 32546414

[27] Zhang L, Fu Y, Wang W, Liu Y, Hu R, Wang Z, et al. Surveillance of adverse events following varicella vaccine immunization in Jiangsu province, China from 2017 to 2023. BMC Infect Dis. 2024;24(1):983. doi: 10.1186/s12879-024-09903-y 39285384 PMC11403869

[28] Gruber MF. US FDA review and regulation of preventive vaccines for infectious disease indications: impact of the FDA Amendments Act 2007. Expert Rev Vaccines. 2011;10(7):1011–9. doi: 10.1586/erv.11.52 21806396

[29] Haber P, Moro PL, Cano M, Vellozzi C, Lewis P, Woo EJ, et al. Post-licensure surveillance of trivalent live-attenuated influenza vaccine in children aged 2-18 years, vaccine adverse event reporting system, United States, July 2005-June 2012. J Pediatric Infect Dis Soc. 2015;4(1):82–3. doi: 10.1093/jpids/piu123 26407365

[30] Sebastian J, Gurumurthy P, Ravi MD, Ramesh M. Active surveillance of adverse events following immunization (AEFI): a prospective 3-year vaccine safety study. Ther Adv Vaccines Immunother. 2019;7:2515135519889000. doi: 10.1177/2515135519889000 31799496 PMC6873273

[31] Liu Y, Lin H, Zhu Q, Wu C, Zhao Z, Zheng H. Safety of Japanese encephalitis live attenuated vaccination in post-marketing surveillance in Guangdong, China, 2005-2012. Vaccine. 2014;32(15):1768–73. doi: 10.1016/j.vaccine.2013.11.107 24503272

[32] Hills SL, Soeung SC, Sarath S, Morn C, Dara C, Fischer M, et al. An evaluation of adverse events following an immunization campaign with the live, attenuated SA14-14-2 Japanese encephalitis vaccine in Cambodia. PLoS One. 2022;17(6):e0269480. doi: 10.1371/journal.pone.0269480 35679297 PMC9182652

[33] Demoly P, Adkinson NF, Brockow K, Castells M, Chiriac AM, Greenberger PA, et al. International consensus on drug allergy. Allergy. 2014;69(4):420–37. doi: 10.1111/all.12350 24697291

